# Influence of beta-cluster haplotypes, alpha-gene status and UGTA1 polymorphism on clinical and hematological data in sickle-cell disease children from French Guiana

**DOI:** 10.1371/journal.pone.0238691

**Published:** 2020-09-03

**Authors:** Narcisse Elenga, Emma Cuadro-Alvarez, Elise Martin, Falucar Njuieyon, Antoine Defo, Chimène Maniassom

**Affiliations:** Pediatric Unit, Cayenne General Hospital, Cayenne, French Guiana; University of Naples Federico II, ITALY

## Abstract

**Objectives:**

This cross-sectional study aimed to investigate the influence of haplotypes, alpha-gene status and UGTA1 polymorphism on the severity of sickle cell disease in children.

**Methods:**

This cross-sectional study was conducted between 2012 and 2014 at the Cayenne Hospital, in French Guiana. Acute clinical complications were grouped into (i) severe SCD defined by the presence of stroke and/or abnormal-transcranial Doppler (TCD), (ii) moderate SCD defined by the presence of at least three annual events requiring hospitalization and/or at least one acute chest syndrome, (iii) no severe SCD (in the absence of the precited events).

**Results:**

Among the 86 patients, 33.7% were female with a median age of 10 years (range: 6–12 years). The vast majority of patients had SCA (HbSS) phenotype (74.4%; n = 64). The severe haplotype was found in 40% of patients. 30% were BEN/BEN. Analysis of α-globin gene deletions revealed that 32 patients (37.2%) were heterozygous (loss of 2 genes in 2 cases and loss of 1 gene in 30 cases) for α-thalassemia (3.7 kb deletion). Homozygous (TA) n TA7/7 was found in 24 (28%). In the multivariate analysis, the factors associated with the severity of sickle cell disease were the first vaso-occlusive crisis before one year of age (OR 25, [95% CI = 6.0–107.0], p<0.001) and a baseline MCV >80 fL (OR 0.20 [95% CI = 0.04–0.96], p = 0.04). The area of the ROC curve was 0.90.

**Conclusion:**

Prospective studies with greater statistical power would provide more knowledge on the relationship between UGT1A1 mutations and the clinical and hematological manifestations of SCA.

## Introduction

French Guiana is an overseas French territory located in South America (capital town: Cayenne), situated on the northeastern coast of South America. French Guiana is bounded by the Amapa state of Brazil to the south and east and the Atlantic Ocean to the north east. The Maroni River forms the French Guiana–Suriname border in the west. The 2015 population in this region was approximately 250,377 people [1]; around 40 percent of the population lives in the capital. The population is composed of people of mixed African and French ancestry (Creoles), with minorities of metropolitan French, Brazilians, Surinamese, Haitians and other Caribbeans, Chinese and Laotians and the Maroon community. Maroons (or Bushinengue) are descendent from African slaves who escaped (marooned) from the plantations of the current Suriname, a Dutch colony at the time of the slave trade and who banded together and build their own settlements.

Sickle cell disease (SCD) is a major public health problem in French Guiana [2, 3]. It is an inherited hemoglobin disorder, characterized by a high level of hemoglobin S, which polymerizes under certain conditions (deoxygenation), thus deforming erythrocytes and leading to the clinical symptoms of this disease. During disease evolution, the β^S^ mutation appeared at least five times in distinct geographical areas, thus leading to five β^S^-globin gene haplotypes that are named according to their country/region of origin: (i) Bantu or Central Africa Region (CAR), Benin (BEN), Senegal (SEN), Arabic-Indian (Saudi) and Cameroon (CAM) [4, 5]. Later on, some atypical β^S^ haplotypes resulting from gene conversion and/or recombination of the five original haplotypes were described [6–8].

Many authors have tried to correlate the clinical severity of SCD with the βS^S^ haplotypes. Despite some contradictory results, it is generally recognized that the Senegal and Arabic-Indian haplotypes are associated with fewer complications because of higher residual HbF levels. However, many studies were conducted on populations with only one or two over-represented β^S^ haplotypes [9, 10]. The very heterogeneous population of French Guiana is thus an excellent opportunity to characterize the role of the β^S^ haplotypes in a single study without any other confounding factor. We also genotyped the alpha-gene status and the thymine-adenine (TA)n UGT1A1 promoter polymorphism since they are recognized as potential SCD genetic modifiers, respectively [11, 12]. The promotor region contains a run of TA repeats, differing in the number of repeats, from 5 to 8. Four alleles are described in humans: (TA)5, (TA)6, (TA)7 and (TA)8. Low activity has been reported in humans with (TA) 7/7, 7/8 and 8/8 and normal or high activity in (TA) 6/6, 5/5, 5/6) [13]. The first part of this study showed that the most frequent β^S^ haplotypes were the Benin haplotype (65.9% of the chromosomes) and the Bantu (20.5%) haplotype. Alpha-thalassemic deletions were present in 37% of the patients and homozygosity for the (TA)7 allele of the UGT1A1 promoter in 21.4% [14]. This second study on children under 15 years old aimed to investigate the influence of haplotypes, alpha-gene status and UGTA1 polymorphism on the severity of SCD in French Guianese children.

## Patients and methods

The study population was a part of a pre-existing cohort, Improving the quality of management of SCD in French Guiana: "Epidemiology of predictive factors of acute clinical events," that enrolled approximately1000 patients. This cross-sectional study was conducted between 1 January 2012 and 31 December 2014, in children under 15 years, with SCD (HbSS, HbS-beta, or HbSC diagnosed by neonatal screening), living in Cayenne and the surrounding area and followed at the Outpatient SCD Center at Cayenne Hospital, in French Guiana, since 2005. The other inclusion criteria in our study were: having the following examinations available in the medical records: beta-cluster haplotypes, alpha-gene status and UGTA1 polymorphism (these three examinations not being carried out routinely, the absence of these in the medical file constituted an exclusion criterion), not being on hydroxyurea (HU) or chronic transfusion. The hemoglobin phenotype was detected through capillary electrophoresis. DNA was extracted from blood leukocytes by silica-membrane-based nucleic acid purification. Alpha-thalassemia was characterized by a single-tube multiplex-PCR genotype assay detecting the -a3.7 and -a4.2 single gene deletions, the—SEA double-gene deletions, and the—MED and 2a20.5 Mediterranean double gene deletions. UGT1A1 polymorphisms were analyzed by a dedicated high resolution melting (HRM) method [15]. The five main SCD haplotypes were identified by genotyping the dedicated HincIIe, XmnI, HindIIIGc, HindIIIAc, HincIId, HincIIwb and AvaII SNPs using specific HRM and FRET Light Cycler methods [16]. Clinical and biological data were collected during the annual monitoring of patient medical records. All these biological parameters were performed at steady state, apart from any VOC, other complication or hospitalization. These biological parameters were also recorded before hydroxyurea (HU) or chronic transfusion introduction. The following laboratory parameters, were analyzed: Hb level, white blood cells (WBC), mean corpuscular volume (MCV ≤80 versus>80 fL) and HbF level (patients with HbF ≥ 20% and those with HbF < 20%). The clinical outcomes were analyzed according to the medical and surgical history obtained from the patient’s medical records. Acute clinical complications were grouped into (i) severe SCD defined by the presence of stroke and/or abnormal-transcranial Doppler (TCD), (ii) moderate SCD defined by the presence of at least three annual events requiring hospitalization and/or at least one acute chest syndrome, (iii) no severe SCD (in the absence of the precited events). Because of the well-known different clinical evolution between the SCA, SC or Sβ-thalassemia genotypes, two groups according severity were created (group 1: severe genotype SCA and Sβ°-thalassemia and group 2 SC and Sβ+-thalassemia). The beta-haplotypes were classified as severe: at least one CAR or BAN, moderate: other haplotypes. The–α^3.7^globin gene deletion status was also classified as: (i) carriers of a single α-globin gene defect (silent trait), (ii) carriers of two α-globin gene defect (α-thalassemia trait). We classified UGT1A1 promoters into two groups: group 1 included (TA) 7/7, 7/8 and 8/8 as they were shown to increase the risk of gallstones in SCD patients [17–19]) and group 2 (TA) 6/6, 5/5, 5/6 with low risk of gallstones. Patients’ medical records were accessed throughout the study period (between 1 January 2012 and 31 December 2014). The anonymous database, based on medical records, was created between1 January 2013 and 30 June 2014.

### Ethical and regulatory aspects

The parents or authorized representatives granted written and informed consent to participate in this research. This study was approved by the "Comité local d'éthique du Centre Hospitalier de Cayenne Andrée Rosemon" (Number 1-2017-V2) which is the only ethics committee in French Guiana likely to authorize any biomedical research, and the database was declared at the Commission Nationale Informatique et Liberté (CNIL, Number3Yj157849 3). Patients were informed of the utilization of their data with an informative poster in the medical units concerned with SCD.

### Statistical analyses

Data were analyzed using STATA 15.0 (Stata Corp LP, College Station, TX, USA) software. The results are expressed as medians ± standards deviations. Fisher’s exact test was used to study categorical variables while the Kruskal Wallis test was used to study non-Gaussian variables. The factors associated with haplotype and alpha gene deletion were analyzed by ordered multiple logistic regression. For all tests performed, a p-value of 0.05 or less was considered as statistically significant. We included in our multivariate model covariates that were associated with the outcome and other factors, according to the medical literature.

## Results

**Fig 1:**
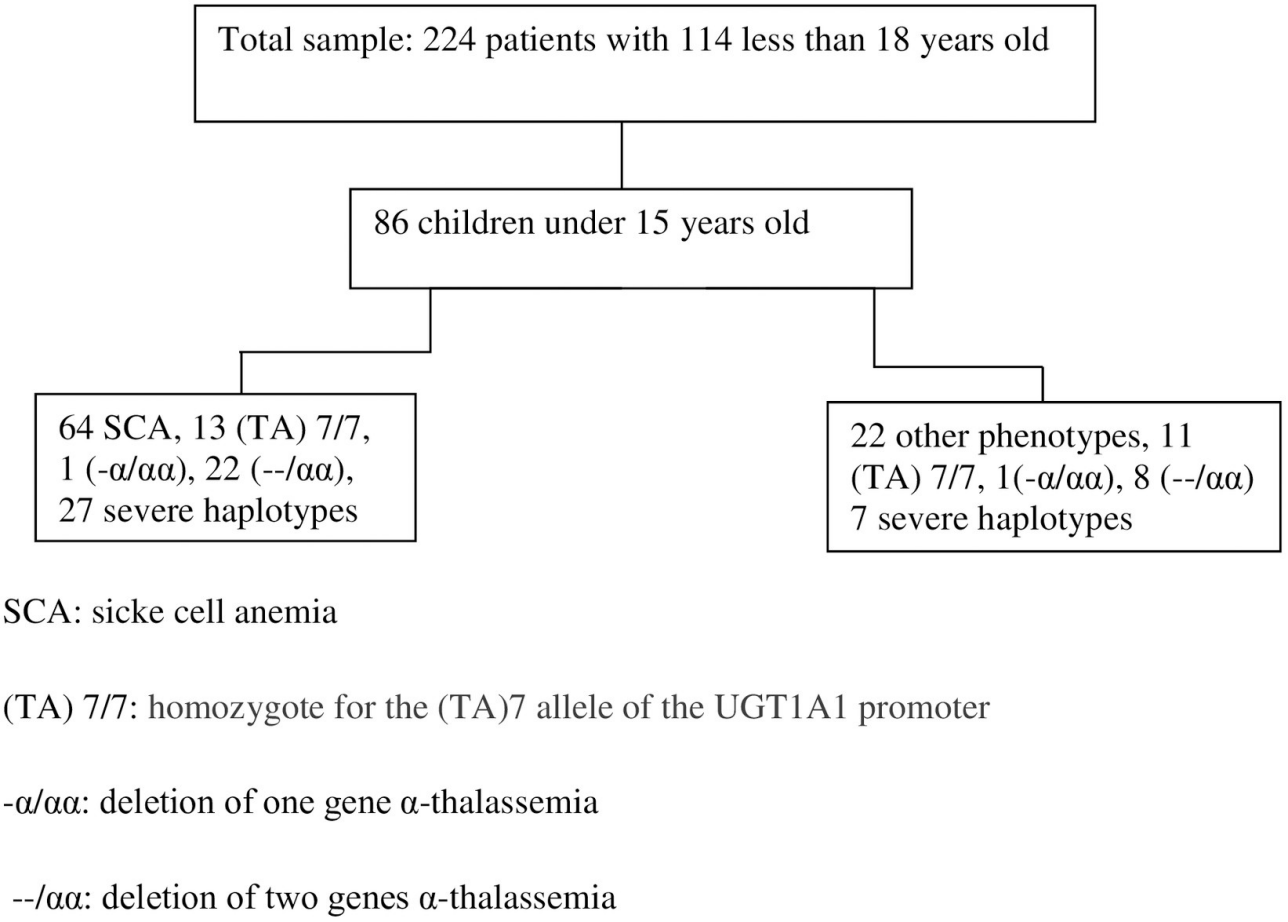
Flow chart of the distribution of the study patients.

The description of the study sample is presented in Fig 1 and Table 1. Among the 86 patients, 33.7% were female with a median age of 10 years (range: 6–12 years). Patients originated mostly from French Guiana (57%; n = 49). All our SCD patients were diagnosed by newborn screening. The vast majority of patients were determined to have SCA (HbSS) (74.4%; n = 64). The severe haplotype was found in 40% of the cohort. 30% were BEN/BEN. Analysis of α-globin gene deletions revealed that 32 patients (37.2%) were heterozygous (loss of 2 genes in 2 cases and loss of 1 gene in 30 cases) for α-thalassemia (3.7 kb deletion). Also, the co-inheritance of α-globin gene deletion and triplication was not found in the studied patients. Homozygous (TA) n TA7/7 was found in 24 (28%). In this cohort, 21 patients (24.4%) were treated with hydroxy-urea (HU) at a median dose of 20 mg/kg per day for at least one year between 2005 and 2015 at a median age of 6 years [6–12].

**Table 1:** Clinical and hematological characteristics and factors associated with severe SCD.

Variables	Severe SCD	Moderate SCD	No severe SCD	p*	AOR	p**
**Age (years), Median, IRQ**	10 [6–12]	12 [7.5–13]	9 [6–12]	0.2		**0.8**
**Sex, Male, n (%)**	5 (5.8)	11 (12.8)	41 (47.7)	0.8		
**1st VOC before age<one year, n (%)**				<0.001	**25 [6–107]**	**<0.001**
***Yes***	8 (89)	14 (87.5)	14 (23)			
***No***	1 (11)	8 (12.5)	47 (77)			
**Origin, n (%)**				0.6		
***Guianese***	5 (56)	10 (62.5)	34 (56)			
***Haitian***	4 (44)	4 (25)	19 (31)			
***Others***	0 (0)	2 (12.5)	8 (13)			
**Alpha thalassemia deletion, n, %**	2 (6)	6 (18)	25 (76)	0.5		
**UGT1A1 alleles, n, %**				0.2		**0.3**
***(TA) 6/6*, *5/6*, *5/7*, *6/7*, *6/8)***	6 (67)	10 (62.5)	46 (75)			
***(TA) 7/7***	3 (33)	6 (37.5)	15 (25)			
**Haplotype, n (%)**				0.7		
***Severe***	3 (33)	6 (37.5)	25 (41)			
***Moderate***	6 (67)	10 (62.5)	36 (59)			
**Genotype, n (%)**				0.5		
***SS***	9 (100)	13 (81)	42 (69)			
***Others***	0 (0)	3 (19)	19 (31)			
**HbF level≥20%, n (%)**	4 (4.7)	5 (5.8)	19 (22.1))	0.7		
**Hb level g/L, Median, IRQ**	9.0 [8.0–9.0]	8.05 [7.55–8.75]	9 [8–10]	0.03		**0.2**
**WBC, G/L, Median, IRQ**	10 [9–14]	12 [10–12]	10 [10–12]			
**MCV, fL, Median, IRQ**	84 [80–90]	85 [80–88,5]	80 [70–87]	0.03	**0.15 [0.18–0.96]**	**0.04**

WBC: White Blood Cells

MCV: Mean corpuscular volume AOR: Adjusted Odds Ratio

VOC: Vasoocclusive crisis, MCV: Mean corpuscular volume, WBC: White blood cells

IRQ: Interquartile range

p*: p-value obtained after univariate logistic regression

p**: p-value obtained after multivariate logistic regression

**Fig 2:**
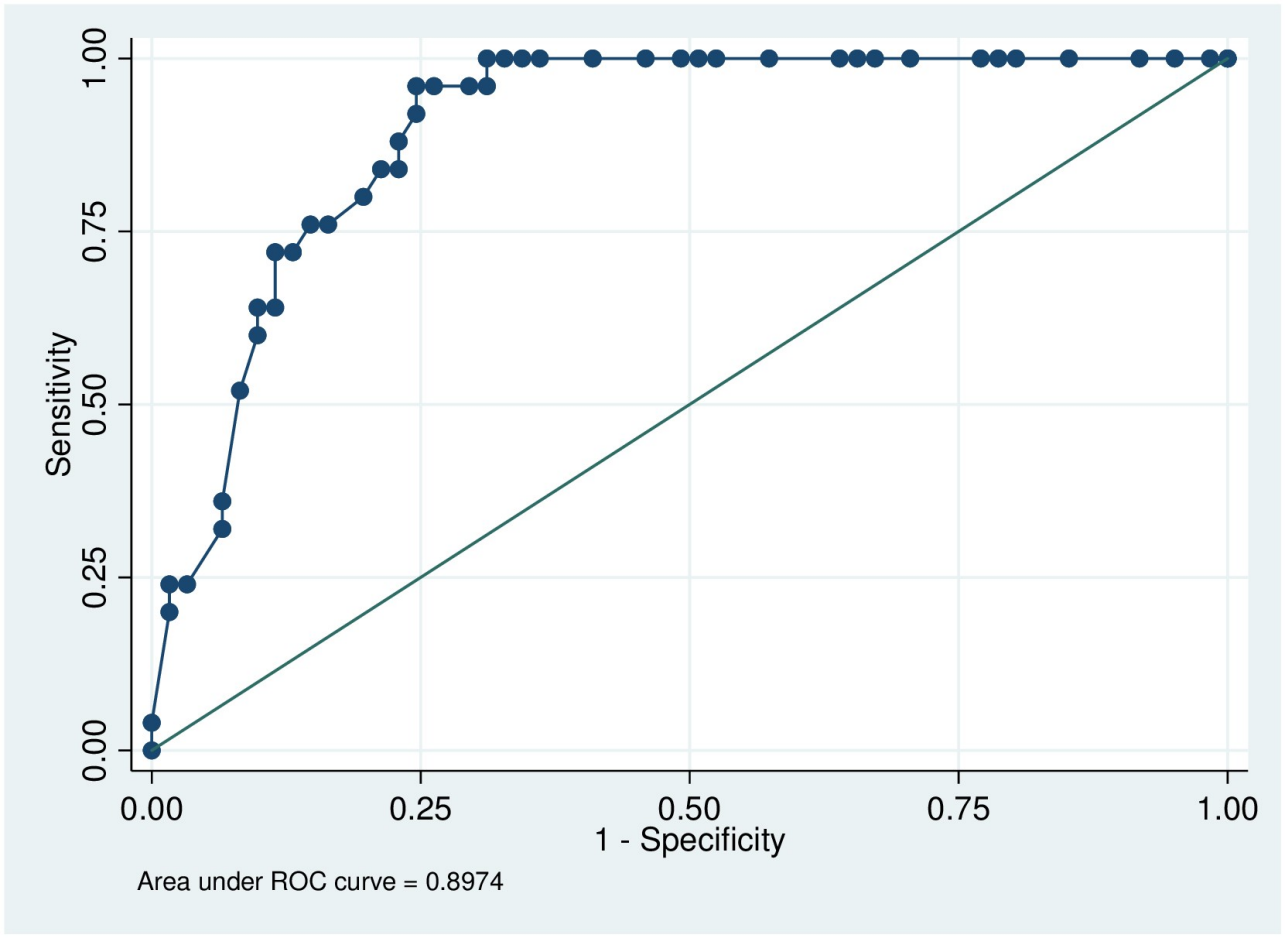
ROC curve testing our multivariate model. The area under the ROC curve was close to 0.9, confirming the quality of the model. ROC analysis was used here to quantify how accurately our medical diagnostic test (the first vasoocclusive crisis before one year of age and the baseline mean corpuscular volume >80 fL) can discriminate between severe and moderate SCD.

In the multivariate analysis, the factors associated with the severity of SCD were the first vaso-occlusive crisis (VOC) before one year of age (OR 25, [95% CI = 6.0–107.0], p<0.001) and a baseline MCV >80 fL (OR 0.20 [95% CI = 0.04–0.96], p = 0.04). The receiver operating characteristic (ROC) analysis was used to quantify how accurately our medical diagnostic test (the first vasoocclusive crisis before one year of age and the baseline mean corpuscular volume >80 fL) could discriminate between severe and moderate SCD. In this multivariate model, the area of the ROC curve was 0.90 (Fig 2). However, we did not observe any significant association between β-globin haplotypes and SCD severity in this cross-sectional study.

## Discussion

This study was conducted to determine the influence of β^S^-globin haplotypes, alpha-gene status and UGTA1 polymorphism on clinical manifestations of SCD. Although the power was insufficient, which could explain the lack of differences in the clinical and hematological characteristics of the studied patients, the data obtained allowed describing the haplotypes present in French Guiana and the prevalence of alpha-thalassemia and UGTA1 polymorphisms in SCD. The BEN type was the most prevalent, followed by the CAR, SEN, CAM and Arab- Indian haplotypes [14]. This distribution of haplotypes was identical to that described in Guadeloupe and Trinidad [20, 21]. However, it differed from those previously described in Brazil [22, 23]. Numerous studies have been published on β^S^-globin haplotypes. They showed a different distribution according to the studied populations. Some of them used this marker as an anthropological tool, while others were interested by its role in the modulation of the expression of the clinical and hematological manifestations of SCD. Despite the low study power, the presence of these markers and their possible influences on the phenotype of SCD patients in a particular population has not been found in other studies [23–29]. This strengthens the notion that, although the genetic event is unique and related to β^S^ gene mutation, the clinical outcome of SCD implies multiple organs and systems as well as complex mechanisms, which are not clearly understood. In this context, there is a high degree of diversity in the prognostic markers, which have not yet been strengthened by the scientific community, except the genetic origin of SCD and the presence of hemoglobin S.

According to the literature [30–32], some sickle cell haplotypes contain genetic modifiers that are associated with increased levels of fetal hemoglobin, which is resistant to sickling. Thus, the CAR haplotype is associated with more severe clinical manifestations, while the SEN, CAM and SAUDI haplotypes present high HbF levels with milder manifestations, and the BEN haplotype presents an intermediate HbF level and clinical course [33]. The impact of β haplotypes was not significant, due probably to the small sample and the young age of the study population. We also studied the association between clinical severity of SCD and alpha-thalassemia. This other genetic disease is known to be an important modifying factor of clinical severity in SCD. The hemoglobin F level was higher in patients with alpha-thalassemia. It has been suggested that alpha-thalassemia reduces the intraerythrocyte HbS concentration and consequently the polymerization of deoxyHbS and hemolysis [33].

Although there have been many studies on the association between alpha-thalassemia and SCD, little is known about the relationship between BS-haplotypes and UGTA1 polymorphisms. UDP-glucuronosyl transferase 1.1, also known as UGT1A1, is an enzyme encoded by the UGT1A1 gene. UGT1A1 is the only enzyme involved in the conjugation of bilirubin. Mutations of this gene cause serious problems with bilirubin metabolism, but the syndrome can be caused by one or more mutations. They differ mostly by symptoms and not particular mutations [34]. UGT1A1 coding region mutations have been linked to Gilbert syndrome in Asian populations, and homozygosity for UGT1A1 is a causative factor for Gilbert’s syndrome [35, 36]. Patients with a UGTA1 mutation have a high level of hemoglobin F. In sickle cell patients, a high concentration of hemoglobin F has a protective role on the appearance of VOC, and therefore the complications of the disease. In fact, some authors [37] consider hemoglobin F as the most important factor expression modifier of SCD. The protective role of UGTA1 mutations has not been previously described. Given the low power of our study, other studies on the protective role of this polymorphism would be useful to test our hypothesis.

The effect of HU on hemoglobin F production is one of its multiple mechanisms of action. HU effectively decreases the frequency of acute complications such as VOCs and ACS, and improves the rheological characteristics of RBCs, as previously reported [38]. However, there are patients with low HbF levels with moderate disease and others with high HbF levels with severe SCD. This implies that the HbF level is not the only determinant of the rheology of RBCs. Other hematological factors such as MCV must be tested to determine the best outcome in terms of the severity of SCD. In our study, MCVs greater than 80 fL appeared to be protective against severe SCD. Indeed, MCV as a factor modulator of SCD severity has rarely been described. Lamarre et al. [39] reported lower MCV in SCA children with acute chest syndrome (ACS). This protective effect of high MCV may not be simply the effect of HU, as treatment with HU significantly increases MCV [40]. A first VOC before the age of one year was found to be a risk factor for severe disease. This result is consistent with past studies describing the association of dactylitis with more severe outcomes [41–43].

## Conclusion

In this study, we demonstrated that the factors associated with the severity of SCD were the first VOC before one year of age. This result is consistent with previous studies. However, a baseline MCV >80 fL also seems to be a protective factor. Prospective studies with greater statistical power would provide more knowledge on the relationship between UGT1A1 polymorphisms and the clinical and hematological manifestations of SCA.

## Supporting information

S1 File (XLSX)
